# Beyond structural MRI: combined diffusion metric improve delineation of epileptogenic tissue in focal cortical dysplasia

**DOI:** 10.1038/s41598-026-57292-w

**Published:** 2026-06-09

**Authors:** David Kala, Timur Abragimovich, Radek Janča, Nora Profantová, Miroslav Koblížek, Adam Kalina, Martin Kudr, Yeva Prysiazhniuk, Lukáš Michal, Jan Šanda, Zuzana Holubová, Kateřina Jiránková, Petr Marusič, Pavel Kršek, Martin Kynčl, Jakub Otáhal

**Affiliations:** 1 Department of Pathophysiology, Second Faculty of Medicine, Charles University, Plzeňská 311, 150 06 Prague 5, Czech Republic; 2 Department of Radiology, Second Faculty of Medicine, Charles University and Motol University Hospital, Prague, Czech Republic; 3 Department of Paediatric Neurology, Second Faculty of Medicine, Charles University and Motol University Hospital, Prague, Czech Republic; 4 Department of Neurology, Second Faculty of Medicine, Charles University and Motol University Hospital, Prague, Czech Republic; 5 Department of Circuit Theory, Faculty of Electrical Engineering, Czech Technical University in Prague, Prague, Czech Republic; 6 Department of Pathology and Molecular Medicine, Second Faculty of Medicine, Charles University and Motol University Hospital, Prague, Czech Republic; 7 Epilepsy Research Centre Prague, EpiReC, Prague, Czech Republic

**Keywords:** Focal cortical dysplasia, Drug-resistant epilepsy, Diffusion MRI, Combined diffusion metric, Epileptogenic tissue, Presurgical mapping, Diseases, Medical research

## Abstract

Accurate delineation of the epileptogenic zone in focal cortical dysplasia (FCD) remains challenging, as structural MRI often underestimates the extent of dysplastic tissue. Diffusion MRI can probe microstructural alterations, yet individual diffusion models provide limited sensitivity and clinical interpretability. We prospectively studied 19 patients with histologically confirmed FCD who underwent presurgical evaluation, surgical resection, and postsurgical MRI. Fourteen diffusion metrics derived from complementary diffusion models were integrated using logistic regression to generate a single combined diffusion metric (CDM). Spatial correspondence among structural MRI, resected tissue, perilesional cortex, and contralateral homologues was assessed using Dice similarity and ROC analyses. Histological cellularity and multimodal imaging data were used for biological validation. Individual diffusion metrics identified microstructural abnormalities within MRI-visible lesions, including reduced anisotropy, lower fibre density, and increased isotropic diffusion. Integration into the CDM improved discrimination between lesional and healthy tissue (*p* < 0.03), detected abnormalities extending beyond radiological borders, and showed spatial concordance with FDG-PET hypometabolism and epileptiform activity in 90% of patients. CDM-guided delineation increased agreement with resected regions and reduced unresected perilesional volume. CDM provides a biologically grounded and clinically interpretable representation of epileptogenic tissue in FCD, refining presurgical mapping beyond conventional MRI and potentially improving surgical targeting.

**Supplementary Information:**

The online version contains supplementary material available at 10.1038/s41598-026-57292-w.

## Introduction

Focal cortical dysplasia (FCD) represents one of the most common causes of drug-resistant focal epilepsy, particularly in children and young adults. Surgical resection remains the only curative treatment option, yet even in patients with well-defined FCD type II lesions, complete seizure freedom is achieved in only about 64–75% of cases^1^. This incomplete success largely reflects the challenge of accurately defining the epileptogenic zone (EZ)—the cortical region capable of initiating seizures, which typically extends beyond the presumed radiological lesion visible on structural MRI. Consequently, a substantial proportion of epileptogenic tissue may remain unresected, leading to persistent seizures despite technically successful operations^2–5^. Accurate delineation of epileptogenic tissue is essential not only for improving surgical outcomes in drug-resistant epilepsy but also for enabling its earlier indication^6^.

Conventional MRI sequences such as T1-weighted, T2-weighted, and FLAIR imaging visualize gross morphological abnormalities such as cortical thickening, blurring of the gray-white matter junction, abnormal signal, or the transmantle sign, that are characteristic but do not encompass all radiological features of dysplastic cortex^7–9^. These techniques primarily capture structural morphology rather than the microstructural disorganization of cortical and subcortical tissue that underlies epileptogenicity. Even with optimized epilepsy protocols, subtle forms of FCD or perilesional abnormalities often remain radiologically silent, limiting the precision of surgical targeting^10–12^.

Diffusion MRI (dMRI) offers a powerful, non-invasive approach to probe tissue microstructure by quantifying the motion of water molecules in vivo. Traditional tensor-based models provide global measures of diffusivity and anisotropy such as mean diffusivity (MD) and fractional anisotropy (FA) but assume Gaussian diffusion within a single compartment and therefore offer only a simplified description of complex neural tissue^13^. In contrast, advanced multi-compartment diffusion models capture complementary aspects of cellular and axonal organization. Diffusion kurtosis imaging (DKI) characterizes non-Gaussian diffusion and tissue complexity, neurite orientation dispersion and density imaging (NODDI) estimates neurite density and dispersion, fixel-based analysis (FBA) provides tract-specific measures of fibre density and morphology, and diffusion basis spectrum imaging (DBSI) separates anisotropic and isotropic diffusion components, distinguishing axonal integrity from cellular or extracellular contributions^14–16^. Recent work from our group demonstrated the feasibility and biological validity of these approaches by revealing microstructural alterations in the thalamus and thalamo-cortical pathways of patients with focal cortical dysplasia using DKI, NODDI and FBA^17^. Comparable applications of advanced diffusion modelling in epilepsy have been reported, highlighting the potential of microstructural imaging for delineating epileptogenic networks beyond visible lesions^18–20^.

These observations underscore a critical translational gap, as advanced diffusion MRI can detect subtle microstructural abnormalities but its practical impact on clinical decision-making remains unclear. Current presurgical workflows often incorporate diffusion MRI for tractography to preserve eloquent pathways, yet diffusion-derived metrics are seldom used to identify epileptogenic tissue or to guide the delineation of the epileptogenic zone. Despite methodological advances, a systematic understanding is lacking of how distinct diffusion models capture epileptogenic microstructure, how these imaging features correspond to histological alterations, and how such information could be rendered clinically interpretable for presurgical decision-making. Addressing this gap requires not only evaluating individual diffusion metrics but also determining whether their integration can generate a single composite metric sensitive to the broader epileptogenic network.

To address these gaps, we examined how advanced diffusion models characterize epileptogenic tissue and whether their combination can enhance clinical interpretation.

We hypothesized that:

i. individual diffusion metrics would reveal distinct microstructural alterations within the radiological lesion visible on structural MRI and its perilesional tissue;
ii. these microstructural changes would correspond to histological features of altered cellularity and cortical disorganization;
iii. individual diffusion metrics would provide limited standalone visual contrast, motivating multivariate integration.; and.
iv. integrating multiple diffusion metrics through a multivariate model, specifically a logistic regression-based combined diffusion metric (CDM), would improve discrimination between pathological and healthy tissue, increase concordance with the resected (epileptogenic) region, and thereby provide tangible added value for surgical decision-making.

By following this progression, from biological specificity to clinical applicability, we aimed to determine whether optimally integrated diffusion information can refine the definition of the epileptogenic tissue beyond what is achievable with structural MRI.

## Results

### Patient characteristics and surgical outcomes

Nineteen patients (age range 6 months − 55 years; mean ± SD = 11.5 ± 13 years; 63% female) with histologically confirmed FCD were included. Sixteen patients were diagnosed with FCD type II and three with FCD type I according to the current international classification. All patients had visible lesion on structural MRI and underwent comprehensive presurgical evaluation followed by resective surgery.

**Fig. 1:**
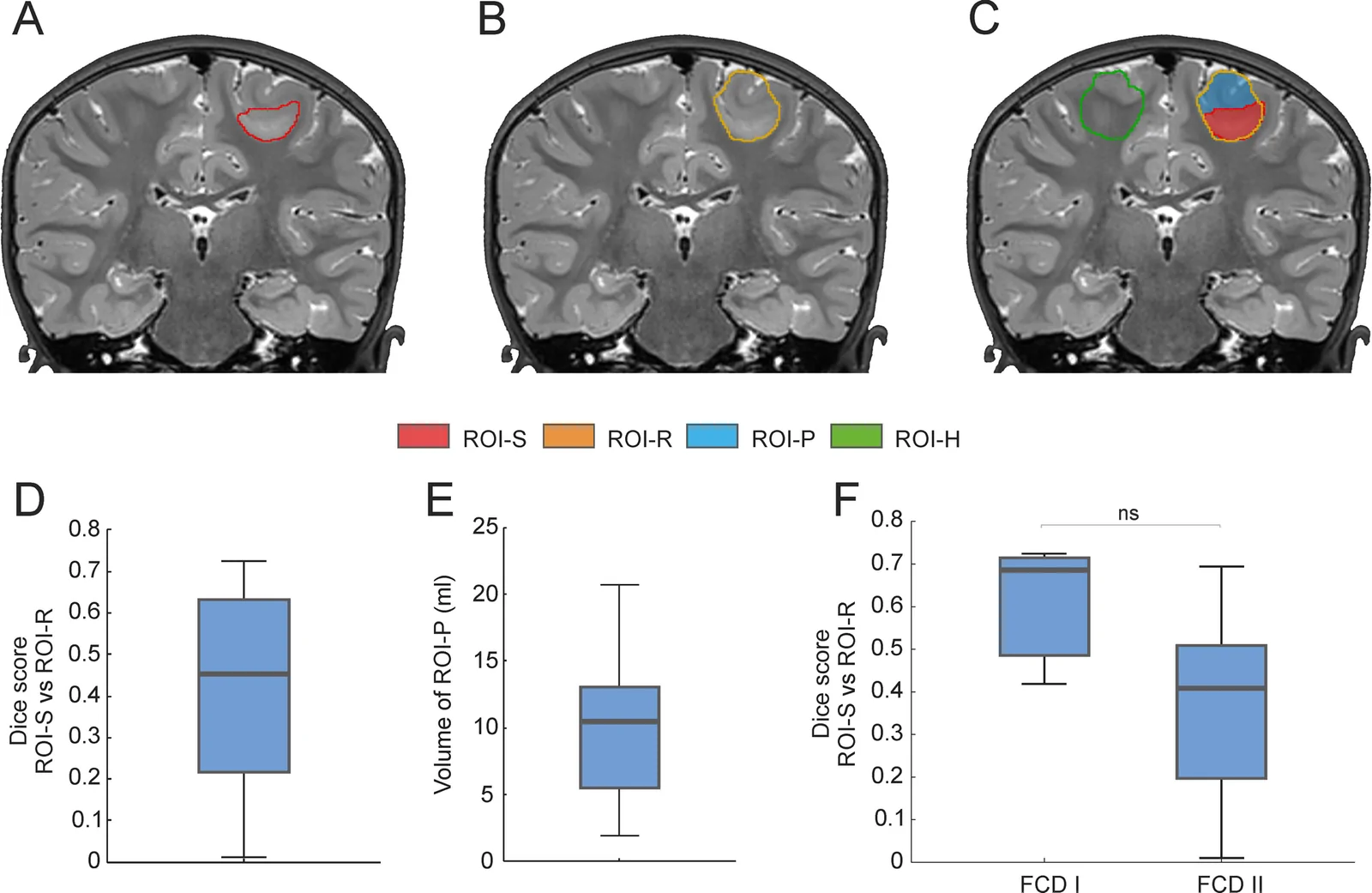
Spatial correspondence between radiological and resected regions in an illustrative patient. Example of patient S7 showing (**A**) the MRI-visible structural lesion (ROI-S), (**B**) the surgically resected region (ROI-R), and (**C**) the combined set of regions including the contralateral homologue (ROI-H) and the perilesional zone (ROI-P). (**D**) Dice similarity coefficients quantify spatial overlap between ROIs, (**E**) boxplot shows perilesional volume, and (**F**) Dice coefficients stratified by histological subtype (FCD I vs. FCD II). The example highlights the limited correspondence between MRI-defined and resected tissue, particularly in FCD II.

Median epilepsy duration was 3 years (IQR = 13 years), and the median seizure frequency was three episodes per day (IQR = 5). Post-surgical outcomes were favourable in most cases: 16 of 19 patients achieved Engel class IA, while three patients (S2, S4, S10) had class IIIA outcomes. In the latter two cases (S2 and S4), seizure frequency was nonetheless reduced by more than 80%. Detailed demographic and clinical characteristics are provided in Supplementary Table 1.

The study design (see Methods, Fig. 6) included systematic delineation of the structural lesion (ROI-S), the resected region (ROI-R), its contralateral homologue (ROI-H), the perilesional tissue (ROI-P), and diffusion-derived abnormalities (ROI-D) for each subject. The median volume of resected tissue was 12.0 ml (IQR = 11.1 ml), and the median completeness of resection, expressed as the proportion of ROI-S removed, was 87% (IQR = 15%). Spatial overlap between ROI-S and ROI-R was only partial, with a median Dice similarity coefficient of 0.38 (IQR = 0.36). The perilesional region (ROI-P), representing tissue resected but not identified radiologically, had a median volume of 6.5 ml (IQR = 13.3 ml). The correspondence between ROI-S and ROI-R appeared higher in FCD I than in FCD II, although this observation was limited by the low number of FCD I cases. An illustrative example of ROI delineation and the spatial relationship between structural and resected regions is shown in Fig. 1.

These findings indicate that although all lesions were radiologically visible, structural MRI underestimated the full extent of epileptogenic tissue later removed during surgery, supporting the initial hypothesis.

### The epileptogenic zone extends beyond the structural lesion

All nineteen patients demonstrated radiologically visible lesions on structural MRI, however, multimodal analyses indicated that the epileptogenic tissue extended beyond the MRI-defined borders. FDG-PET revealed cortical hypometabolism involving or surrounding the structural lesion in 18 of 19 patients. Within the structural lesion (ROI-S), metabolism was fully decreased in eight cases, partially decreased in six, and normal in two, while one patient showed focal hypermetabolism associated with continuous interictal epileptiform discharges.

Perilesional tissue (ROI-P), defined as the surgically resected area not recognised radiologically, showed full or partial hypometabolism in 13 patients and minimal or no change in the remaining six, indicating that metabolic alterations extended beyond the structural lesion. Electrophysiological findings supported this spatial pattern, intraoperative ECoG revealed persistent interictal activity beyond the structural lesion in 14 of 19 patients, and long-term SEEG, performed in three cases, confirmed seizure-onset zones exceeding the MRI-visible abnormality. Post-resection ECoG recordings showed disappearance or marked reduction of epileptiform discharges in all patients who achieved Engel class IA outcomes.

Consistent with these observations, abnormalities identified using the combined diffusion metric also tended to extend beyond the structural lesion, frequently overlapping with hypometabolic regions identified by FDG-PET. Overall, CDM-defined abnormalities showed spatial correspondence with FDG-PET hypometabolism in nearly 90% of patients (see also Supplementary Table 1).

Taken together, these multimodal findings indicate that tissue considered clinically relevant for seizure generation frequently extends beyond the structural lesion identified on MRI, encompassing metabolically and electrophysiologically abnormal cortex that was subsequently included in the surgical resection.

### Diffusion alterations correspond to histological alterations

To assess whether diffusion alterations reflect underlying cellular pathology, we focused on metrics derived from DBSI, a model designed to disentangle anisotropic and isotropic diffusion components and therefore sensitive to changes in tissue cellularity and extracellular space. Quantitative histological data were available in 12 patients, enabling direct comparison between DBSI metrics and quantitative measures of cortical and subcortical cell density within the structural lesion (ROI-S). Regions smaller than 0.5 ml were excluded from the analysis.

**Fig. 2:**
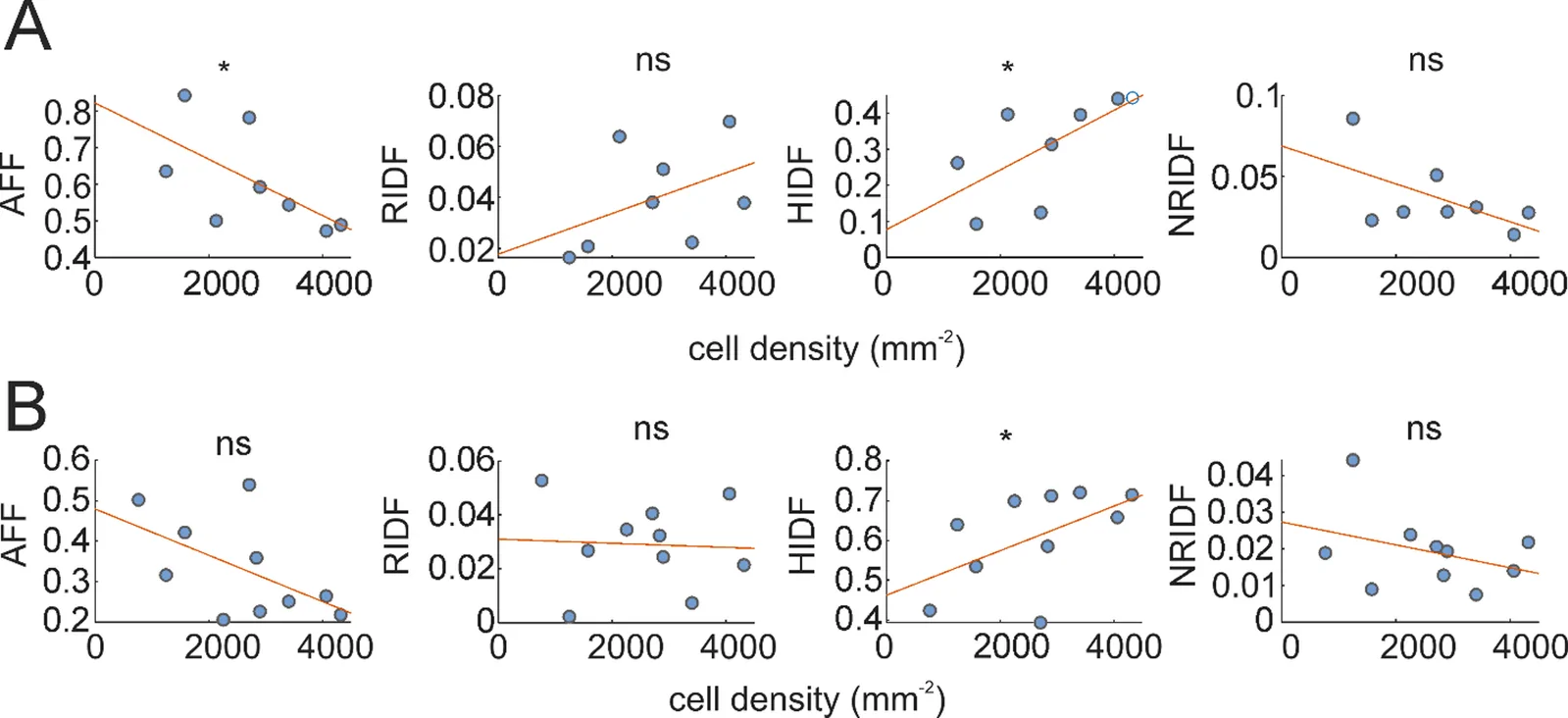
Correlation between DBSI diffusion metrics and histological cellularity. Scatterplots show correlations between DBSI-derived metrics and quantitative cell density measured in the resected tissue. (**A**) In the white-matter subregion of the structural lesion (ROI-S), cellularity correlated negatively with the axonal fibre fraction (AFF) and positively with the hindered isotropic diffusion fraction (HIDF). (**B**) In grey matter, cellularity showed a positive association with HIDF only. The consistent opposite trends between AFF and HIDF indicate that DBSI metrics capture complementary aspects of microstructural disorganisation and cellular heterogeneity within dysplastic cortex.

Median cellularity across all samples was 2772 cells/mm^2^ (IQR = 1487). The FCD I subgroup showed higher cellularity (3862 cells/mm², IQR = 913) than FCD II (2482 cells/mm^2^, IQR = 1322), but this difference did not reach statistical significance (*p* = 0.06). In white matter (Fig. 2A), cellularity correlated negatively with the axonal fibre fraction (AFF; *r* = − 0.62, *p* = 0.045) and positively with the hindered isotropic diffusion fraction (HIDF; *r* = 0.44, *p* = 0.036). In grey matter (Fig. 2B), only HIDF showed a positive correlation with cellularity (Spearman’s *r* = 0.54, *p* = 0.03). None of these correlations survived FDR correction, yet all displayed consistent directional trends.

Subtype analysis revealed that FCD II lesions, which tended to have lower cellularity, exhibited higher AFF and lower HIDF values compared with FCD I (Supplementary Fig. 3). In ROI-P, AFF was significantly higher in FCD II (median = 0.30, IQR = 0.09) than in FCD I (median = 0.23, IQR = 0.02; *p* = 0.023), while a similar but non-significant trend was observed in ROI-S (FCD II: median = 0.37, IQR = 0.17; FCD I: median = 0.27, IQR = 0.03; *p* = 0.10). HIDF values were lower in FCD II than in FCD I both in ROI-S (FCD II: median = 0.58, IQR = 0.17; FCD I: median = 0.67, IQR = 0.03; *p* = 0.06) and ROI-P (FCD II: median = 0.65, IQR = 0.08; FCD I: median = 0.69, IQR = 0.04; *p* = 0.10). These findings mirror the opposite relationships of AFF and HIDF with cellularity and suggest that the reduction in cellular density observed in FCD II is accompanied by relatively preserved anisotropic diffusion. Taken together, these results indicate that DBSI metrics capture microstructural disorganisation and heterogeneous cellular packing characteristic of dysplastic cortex. The opposite behaviour of AFF and HIDF supports their complementary sensitivity to axonal integrity and gliocellular proliferation, linking diffusion abnormalities to the histological substrate of FCD.

**Fig. 3:**
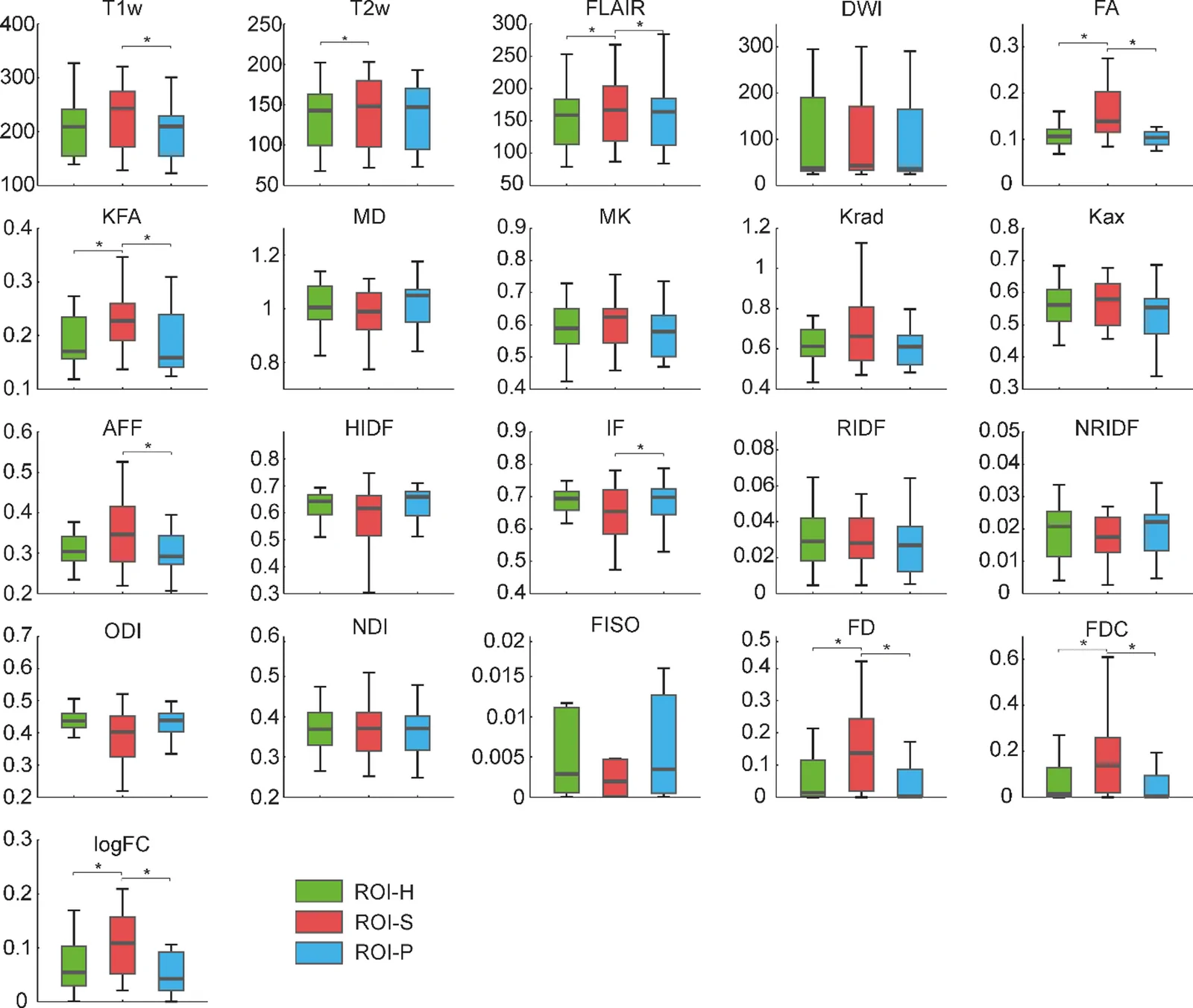
Comparison of structural MRI and diffusion metrics between regions of interest. Group-level comparison of quantitative MRI metrics between the structural lesion (ROI-S), its contralateral homologue (ROI-H), and perilesional tissue (ROI-P). Boxplots show median ± interquartile range for structural (T1w, T2w, FLAIR) and diffusion-derived metrics (DTI, DKI, NODDI, FBA, DBSI). Only a limited subset of diffusion metrics (fractional anisotropy (FA), kurtosis fractional anisotropy (KFA), fibre density (FD), and fibre cross-section (FC)) differed significantly between ROI-S and ROI-H after FDR correction (Supplementary Table 5). The figure illustrates that, while structural MRI consistently delineated the lesion, individual diffusion maps provided only subtle or variable contrast.

### Diffusion abnormalities are visually subtle on individual maps

The visibility of epileptogenic lesions was assessed qualitatively by an experienced neuroradiologist (MKy) across structural and diffusion modalities. Structural MRI sequences (T1w, T2w, and FLAIR) showed clear lesion visibility in all 19 patients, consistent with the inclusion criteria. In contrast, single diffusion maps revealed the lesion only in a subset of cases, and the visual conspicuity varied markedly across models. Among diffusion-derived metrics, mean diffusivity, mean kurtosis, neurite density index, and fibre density occasionally indicated the presence of epileptic tissue, but the margins were indistinct and less sharply defined than on structural MRI. Visual scoring confirmed this impression, the average visibility rating (on a 4-point scale) was 3.9 ± 0.3 for structural MRI, 2.3 ± 0.6 for DTI- and DKI-based maps, and 2.1 ± 0.5 for NODDI- and FBA-based maps. Individual patient- and metric-level ratings are summarised in Supplementary Table 4.

As illustrated in Fig. 3, comparison of structural MRI and diffusion maps demonstrated that diffusion abnormalities were generally less conspicuous, even when localized within the lesion core. Quantitative analysis supported this impression, showing that diffusion signal alterations were present but visually subtle compared with the contrast achieved by structural sequences.

These findings suggest that individual diffusion metrics provide limited standalone visual conspicuity and may be challenging to interpret clinically without integrative analysis.

### Changes on individual diffusion metrics are limited to the structural lesion

To quantify the spatial extent of diffusion alterations, median values from the structural lesion (ROI-S) were compared with those from the perilesional zone (ROI-P) and the contralateral homologue (ROI-H). Group-level analysis demonstrated that most diffusion metrics differed significantly between ROI-S and ROI-H but not between ROI-P and ROI-H, indicating that abnormal diffusion was largely confined to the structural lesion.

As shown in Fig. 3, fractional anisotropy (FA), kurtosis fractional anisotropy (KFA), fibre density (FD), fibre density and cross-section (FDC), and log-transformed fibre cross-section (logFC) exhibited the strongest lesion-related changes (Supplementary Table 5). In contrast, mean diffusivity (MD), mean kurtosis (MK), neurite density index (NDI), and other advanced diffusion metrics showed no significant differences after FDR correction. The pattern of alterations consistently reflected reduced anisotropy and fibre density within ROI-S, consistent with disruption of white matter organisation and partial loss of axonal coherence. Perilesional tissue (ROI-P) demonstrated intermediate values for most metrics but did not differ significantly from the contralateral homologue region, suggesting preserved microstructural integrity despite proximity to the lesion margin. Subgroup analysis revealed similar tendencies in both FCD I and FCD II, although FCD II cases showed slightly more pronounced reductions in FA and FD.

These findings indicate that the majority of diffusion abnormalities are restricted to structural lesion itself, with surrounding tissue showing diffusion properties comparable to healthy cortex.

### The combined diffusion metric enhances delineation of epileptogenic tissue

To improve sensitivity for detecting microstructural abnormalities, all diffusion parameters were integrated into a single logistic-regression-based combined diffusion metric (CDM). The model coefficients were derived from ROI-based data by maximising discrimination between the structural lesion (ROI-S) and its contralateral homologue (ROI-H). The relative contribution of individual metrics decreased in the following order: FA > NDI > ODI > NRIDF > logFC > HIDF > MD > MK > RIDF > FD > FISO > KFA > AFF ≈ IF (full coefficients are provided in Supplementary Fig. 4). When the model was retrained using the resected region (ROI-R) instead of ROI-S, no significant change was observed either in the learned coefficients or in the resulting correlation strength (*p* = 0.17). Similarly, training restricted to FCD II patients yielded comparable results (*p* = 0.51), confirming the stability of the model across subgroups.

**Fig. 4:**
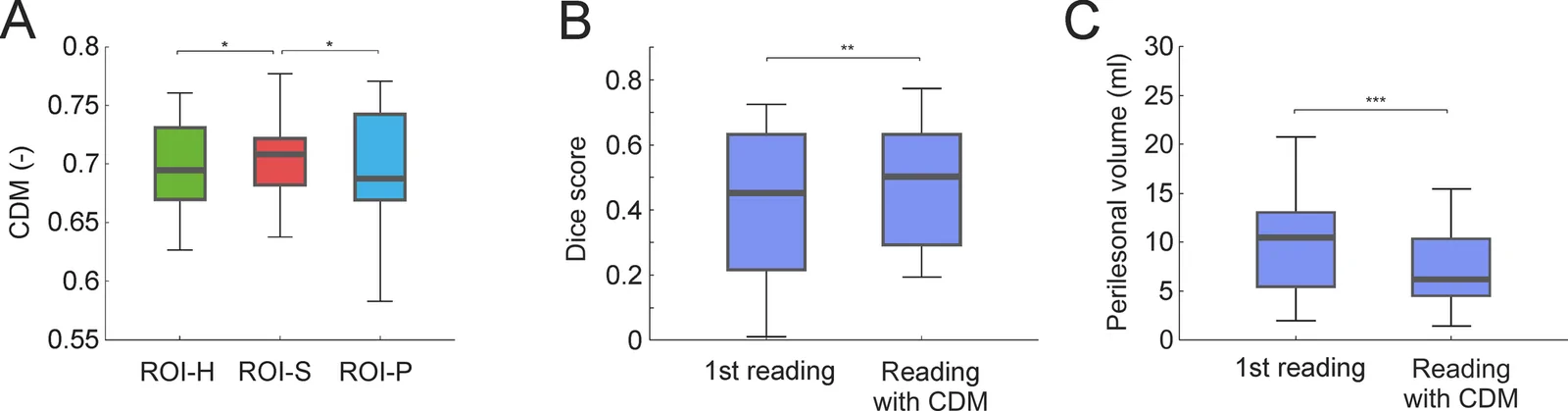
Combined diffusion metric improves delineation of epileptogenic tissue. (**A**) Group comparison of CDM values showing significant differences between the structural lesion (ROI-S) and both the contralateral homologue (ROI-H) and the perilesional region (ROI-P). (**B**) Radiological delineation guided by CDM achieved higher spatial correspondence with the resected region (ROI-R) compared with the initial MRI-based reading, as quantified by the Dice coefficient. (**C**) Use of CDM also reduced the perilesional volume, defined as tissue resected but not identified radiologically. Together, the panels illustrate that CDM highlights diffusion abnormalities extending beyond the structural lesion and improves agreement between imaging and surgical findings.

The CDM showed lesion-related differences between pathological and reference regions. The median correlation coefficient (R) derived from the Mann–Whitney U-test was 0.22 (IQR = 0.24) for CDM, compared with 0.11 (IQR = 0.14) for FA, 0.16 (IQR = 0.29) for NDI, 0.07 (IQR = 0.18) for ODI, and 0.25 (IQR = 0.21) for MK (Fig. 4A). Median CDM values differed significantly (after FDR correction) between ROI-S and both ROI-H (*p* = 0.030) and ROI-P (*p* = 0.024), while ROI-H and ROI-P did not differ (*p* = 0.68). The corresponding medians were 0.71 (IQR = 0.04) in ROI-S, 0.69 (IQR = 0.06) in ROI-H, and 0.68 (IQR = 0.08) in ROI-P. These findings indicate that CDM captures lesion-specific diffusion alterations comparable to, or higher than, most individual diffusion metrics.

To evaluate potential clinical impact, neuroradiologist (MKy) re-delineated the epileptogenic lesion using the structural MRI and ROI-S mask together with the CDM map as guidance. The second reading achieved a significantly higher spatial correspondence with the resected region (ROI-R) compared with the original MRI-based delineation (Dice coefficient: median = 0.50, IQR = 0.31 vs. 0.45, IQR = 0.42; *p* = 0.003; Fig. 4B). The perilesional volume, defined as tissue resected but not radiologically identified, decreased from 10.5 ml (IQR = 7.5 ml) to 6.1 ml (IQR = 6.0 ml; *p* < 0.001; Fig. 4C). The improvement was most pronounced in patients with FCD II, while trends in the same direction were observed for FCD I, albeit without statistical significance due to the small subgroup size.

**Fig. 5:**
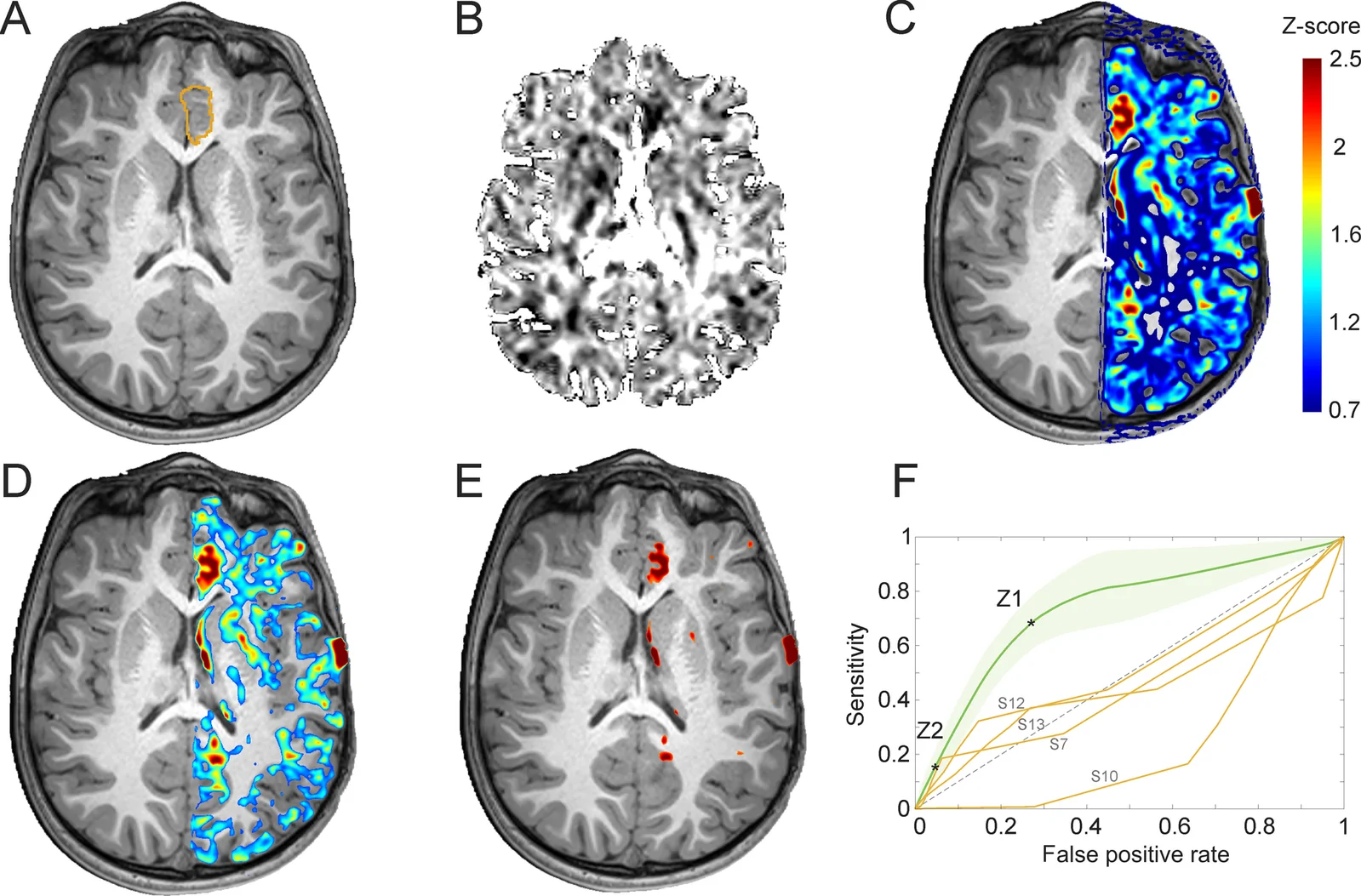
Example of Z-score calculation and ROC-based thresholding in a representative patient (S5). (**A**) T1w MRI showing the resected region (ROI-R). (**B**) CDM map. (**C**) Z-score map representing the statistical difference between the ipsilateral hemisphere and a template derived from the contralateral hemispheres of all patients. (**D**,**E**) Thresholded Z-score maps illustrating voxels exceeding Z > 1 and Z > 2, respectively. (**F**) ROC analysis. The green curve represents the mean ROC with the standard deviation shown as shading. Four patients (S7, S10, S12, S13) deviated from the rest of the dataset and are shown separately in orange. Markers Z1 and Z2 indicate the average position on the ROC curve corresponding to Z-score thresholds of 1 and 2, respectively.

We further examined whether CDM could be used for statistical lesion detection. ROC analysis using the resection mask as reference showed limited performance when simple intensity thresholding was applied (AUC < 0.5). Therefore, Z-score maps were generated by comparing each patient’s CDM with a hemispheric template constructed from contralateral hemispheres. This approach improved discrimination sensitivity, yielding an average AUC of 0.66 ± 0.15 for the full cohort and 0.73 ± 0.07 when outlier patients (S7, S10, S12, S13) were excluded (Fig. 5). Outliers were identified post hoc based on markedly divergent individual ROC behaviour and were excluded only in a sensitivity analysis. These patients did not differ from the remaining cases in sex, age, FCD subtype, or lesion volume. Notably, patient S10, who had the lowest AUC, also showed an unfavourable surgical outcome (Engel III A), suggesting that the true epileptogenic zone may have extended beyond the resected tissue. Patient S7 had ongoing focal status epilepticus at the time of MRI acquisition, which may have influenced diffusion-derived measures. No clear common clinical or anatomical feature was identified for S12 and S13.

These findings demonstrate that the logistic-regression-derived CDM enhances delineation of epileptogenic tissue by integrating complementary diffusion features. The metric improves both quantitative discrimination and radiological delineation, offering a reproducible framework for identifying subtle cortical dysplasias and supporting surgical targeting.

## Discussion

In this study, we demonstrate that integrating multiple diffusion MRI models into a single combined diffusion metric (CDM) provides a more comprehensive and biologically meaningful representation of epileptogenic tissue in FCD than any single diffusion metric. Individual diffusion metrics revealed heterogeneous but consistent microstructural alterations within the structural lesion, reflecting cortical disorganisation and altered cellular composition, whereas the integrated CDM identified abnormalities extending beyond the radiological borders. Importantly, the CDM improved the spatial correspondence with surgically resected regions and reduced the extent of perilesional tissue that otherwise appeared structurally normal on conventional MRI, supporting the complementary role of diffusion-derived microstructural features in defining the epileptogenic zone. Extending prior studies on advanced diffusion models^18,21,22^, our results show that multivariate integration of diffusion metrics enhances the delineation of epileptogenic tissue and improves concordance with surgically verified lesions, offering added value for presurgical evaluation in patients with FCD^23^.

The observed associations between DBSI-derived diffusion metrics and quantitative histology indicate that the abnormalities reflect genuine microstructural pathology rather than macroscopic confounds. DBSI separates anisotropic and isotropic components, distinguishing axonal injury from cellular inflammation, oedema and demyelination. Its methodological foundations and histological validation are well established^24,25^ In our cohort, reduced axonal fibre fraction co-occurs with higher histological cellularity, while increased HIDF reflects cellular packing and gliosis, patterns consistent with cortical disorganisation and axonal loss. The opposing behaviour of AFF and HIDF underscores their complementary sensitivity to anisotropic (axonal) versus isotropic (cellular or gliotic) compartments, aligning with previous diffusion-histology studies^21,26^. To our knowledge, this is the first application of DBSI to FCD, providing initial evidence that this technique can non-invasively capture the complex microstructural heterogeneity of the dysplastic cortex. Moreover, subtype specific tendencies observed in our data are concordant with recognised histopathological distinctions between FCD types I and II within the current ILAE classification. ^27,28^. All together, these findings support the concept that advanced diffusion modelling extends histopathological insight to the whole-brain level, enabling in-vivo mapping of epileptogenic microstructural alterations beyond the reach of conventional imaging^22^.

Although individual diffusion maps were often visually inconspicuous, quantitative analyses consistently revealed microstructural abnormalities within the epileptogenic lesion. This reflects the complexity of diffusion-derived contrast, which integrates multiple compartmental contributions and is therefore poorly captured by visual inspection but becomes informative when metrics are integrated or analysed quantitatively. Previous studies using multi-compartment models or multimodal data have similarly shown lesion-specific alterations even in MRI-negative cases, supporting systematic inclusion of quantitative diffusion analysis in presurgical workflows^21,29,30^. Diffusion MRI thus complements structural imaging by providing objective, quantifiable evidence of microstructural pathology beyond visible lesions^31^.

The CDM developed in this study integrated multiple diffusion-derived metrics using logistic regression to generate a single quantitative index optimised to distinguish lesional from healthy tissue. Logistic regression provides a robust and interpretable framework to combine several metrics, assigning each a weight that reflects how strongly it contributes to identifying abnormal tissue^32^. This transparent weighting enables biological interpretation and facilitates model generalisation across heterogeneous datasets^33^. In our cohort, the highest weights were assigned to FA, NDI, and ODI, metrics sensitive to axonal organisation and neurite complexity, whereas MD, MK, and isotropic components (HIDF, NRIDF) showed smaller but complementary importance. This weighting pattern aligns with known histopathological finding of FCD, dominated by cortical disorganisation and altered neurite density predominantly over pure diffusional or oedematous changes^21,27^.

The performance of CDM should be interpreted at two complementary levels. First, in the ROI-based analysis, which compared value distributions between predefined lesional and reference regions, CDM showed lesion-related differences with an effect size within the range of the strongest individual diffusion metrics. This indicates that the integrated metric preserved sensitivity to the diffusion abnormalities captured by individual models. Second, in the spatial radiological analysis, CDM provided added value by translating these distributed diffusion changes into a single map that could guide lesion delineation. CDM-guided delineation increased spatial agreement with the resected region and reduced the perilesional volume not identified on structural MRI, suggesting that the main practical benefit of CDM lies in improving anatomical delineation rather than simply maximizing ROI-based separation. In our cohort, this benefit was observed without an apparent dependence on lesion size, depth, or cortical location; given the size and composition of the cohort, this observation does not formally exclude such effects, and a systematic assessment will require a dedicated analysis in a larger sample. These findings should be interpreted within the context of a standardized 3T epilepsy MRI protocol with multi-shell high b-value diffusion acquisition sufficient for estimating the advanced diffusion models (DBSI, NODDI, DKI) used in CDM; lower b-values or single-shell acquisitions would primarily compromise the estimation of the isotropic compartments contributing to the metric. Together, these findings supports the view that CDM is best interpreted as a complementary delineation tool rather than a standalone threshold-based lesion detector.

The false-positive findings observed in the ROC analysis further highlight that CDM should not be interpreted as a standalone detector of the epileptogenic zone. Similar to other presurgical modalities, CDM identifies one type of abnormality, in this case diffusion-derived microstructural alteration, which must be interpreted together with structural MRI, PET, electrophysiology, and clinical data to define the most likely surgical target. Some CDM-positive regions outside the resection mask may therefore represent false positives, whereas others may reflect abnormal but unresected tissue not captured by the surgical reference. Future approaches combining CDM with other imaging and electrophysiological markers, anatomical priors, spatial regularization, and machine-learning-based classification may help distinguish true clinically relevant abnormalities from spurious detections and improve specificity.

The CDM maintained consistent performance when trained on alternative reference regions (ROI-R instead of ROI-S) or restricted to FCD type II cases, with stable coefficients and effect sizes. This consistency indicates that the weighting scheme captures genuine and reproducible microstructural differences rather than overfitting individual lesions. The recurrent pattern of reduced anisotropy, lowered neurite density, and increased isotropic diffusion appeared across patients and aligns with prior descriptions of cortical disorganisation in dysplastic cortex^22^. Similar integrative frameworks have proven successful in other neurological diseases such as Alzheimer’s disease^34^, glioblastoma^35^, or subtle cortical dysplasias^36^, supporting the translational potential of this biologically interpretable CDM model for presurgical localisation in FCD.

Accurate localisation of dysplastic cortex is known to be a major determinant of postoperative seizure freedom, particularly in FCD where subtle lesion borders often complicate surgical targeting^8,37^. By revealing microstructural abnormalities invisible on conventional MRI, the CDM can refine delineation of the epileptogenic zone and guide more accurate planning of SEEG trajectories and surgical margins. In our cohort, improved Dice overlap between CDM-defined abnormalities and the resected region was associated with favourable Engel class I outcomes, whereas incomplete coverage corresponded with suboptimal seizure control. Notably, the outlier identified in the ROC analysis, characterised by low AUC values, belonged to this group, supporting the interpretation that insufficient spatial delineation of the epileptogenic tissue contributed to the persistence of seizures. These cases represented clinically complex scenarios in which resection was either intentionally limited due to lesion extent or functional constraints, or residual epileptogenic tissue was clinically suspected, further supporting the concept that incomplete coverage of the epileptogenic zone rather than surgical failure per se may underlie persistent seizures. Integrating CDM maps into existing neuronavigation and multimodal fusion platforms could therefore enhance targeting and reduce the risk of incomplete resection. Similar multimodal approaches, in which quantitative diffusion, metabolic, perfusion or electrophysiologic maps are co-registered to stereotactic planning frameworks, have already improved surgical precision and postoperative outcomes in drug-resistant epilepsy^7,15,17,38–43^.

Status epilepticus (SE) represents an additional clinical factor that may contribute to variability in diffusion-derived measures. Gennari et al.^44^ has shown that patients with a history of SE may exhibit altered diffusion parameters, particularly FA, potentially reflecting transient microstructural changes associated with prolonged epileptic activity. These effects have been interpreted as modifiers of diffusion signal rather than primary determinants of imaging findings. In our cohort, five patients had a history of SE prior to MRI (see Supplementary Table 1), with intervals ranging from the same day to approximately three years before imaging. This variability in temporal proximity introduces potential biological heterogeneity within the dataset. Notably, one patient (S7) experienced continuous focal SE at the time of MRI acquisition and was identified as an outlier in the ROC analysis, suggesting that ongoing epileptic activity may have influenced diffusion measurements in this specific case. Taken together, these observations indicate that SE may contribute to inter-individual variability in selected cases, while the overall group-level diffusion patterns are unlikely to be primarily driven by this factor.

Several methodological considerations should be noted. One important consideration relates to the heterogeneous age distribution of our cohort. In line with prior literature, we observed that very young children with incomplete myelination exhibited different diffusion metrics in both healthy tissue and FCD lesions compared with older participants, consistent with known developmental effects of brain maturation on diffusion-derived parameters. However, when diffusion measures were evaluated using within-subject paired comparisons between lesion, perilesional tissue, and contralateral regions, the age-related variability in absolute values was not reflected in the relative diffusion contrasts central to our analyses. This pattern suggests that diffusion properties within the lesion follow the same maturational trajectory as the surrounding healthy tissue. Importantly, the integration of diffusion information into the radiological assessment using the CDM did not show systematic clustering or deviation among younger children compared with older patients. Together, these findings indicate that, although myelination influences baseline diffusion properties, the CDM primarily captures microstructural abnormalities specific for FCD lesion rather than global maturational differences.

Beyond developmental effects, additional sources of biological variability may also influence diffusion measurements across patients. In particular, modelling with logistic regression assumes broadly consistent diffusion alterations across patients, whereas focal cortical dysplasia represents a heterogeneous group of lesions with subtype-specific microstructural features that may differ^27,28^. For example, FCD type II lesions more frequently include a transmantle component extending into white matter, which may increase the white-matter proportion within ROI-S compared with FCD type I and thereby influence diffusion metrics sensitive to white-matter microstructure. While our framework facilitates interpretability, it may average out inter-individual differences in diffusion patterns. Future studies could employ non-linear approach targeting patient-specific heterogeneity. The modest cohort size and predominance of type II lesions limit the generalisability of the diffusion–histology correlations, and more systematic tissue sampling may allow better correspondence between diffusion metrics and histological measures in future studies. Minor registration inaccuracies between MRI and surgical masks may also have influenced overlap estimates^18^. Despite these factors, the consistent results across training schemes supports the robustness of the CDM and its potential for validation in larger, multicentre studies.

Future work should focus on translating the CDM from a research approach into a clinically applicable tool. Although our data do not allow a definitive assessment of MRI-negative or visually occult FCD, the exploratory improvement observed also in FCD I cases suggests that CDM may help in poorly demarcated lesions by providing additional microstructural contrast to support radiological interpretation. Larger cohorts, including MRI-negative or radiologically ambiguous cases, will therefore be essential to assess its diagnostic sensitivity for subtle cortical dysplasias. Combining diffusion-based microstructural information with metabolic and electrophysiological data, such as FDG-PET or SEEG, may further enhance its added value. In parallel, the use of advanced non-linear integration methods and the creation of normative CDM atlases could enable automated, real-time lesion probability mapping in clinical workstations, supporting more accurate and individualised surgical planning.

Our results demonstrate that integrating multiple diffusion MRI metrics into a combined diffusion metric (CDM) provides a more comprehensive representation of epileptogenic tissue in FCD than any single metric alone. Individual diffusion metrics revealed heterogeneous but consistent microstructural alterations corresponding to histological markers of increased cellularity and cortical disorganisation. Although these changes were often visually inconspicuous, the integrated CDM enhanced tissue discrimination, delineated abnormalities beyond the structural lesion and improved spatial concordance with the resected region. Together, these findings indicate that optimally integrated diffusion information captures subtle but clinically meaningful abnormalities beyond conventional MRI, and that incorporating the CDM into multimodal presurgical planning may support more accurate surgical targeting. Its potential impact on postoperative seizure outcome should be evaluated prospectively in larger cohorts.

## Materials and methods

### Study design and ethical approval

This prospective single-centre study was conducted at Motol University Hospital (Prague, Czech Republic) between 2021 and 2024. All patients were recruited within a grant-supported project focused on developing advanced MRI biomarkers in epilepsy. The study was performed in accordance with the Declaration of Helsinki and approved by the institutional Ethics Committee (EK-875.1.42/20). Informed consent for participation, data collection, and publication of anonymized data was obtained from all participants or their legal guardians. A subset of patients was included in a related diffusion MRI study of thalamic pathways^17^, whereas here we focus on cortical and perilesional diffusion abnormalities.

**Fig. 6:**
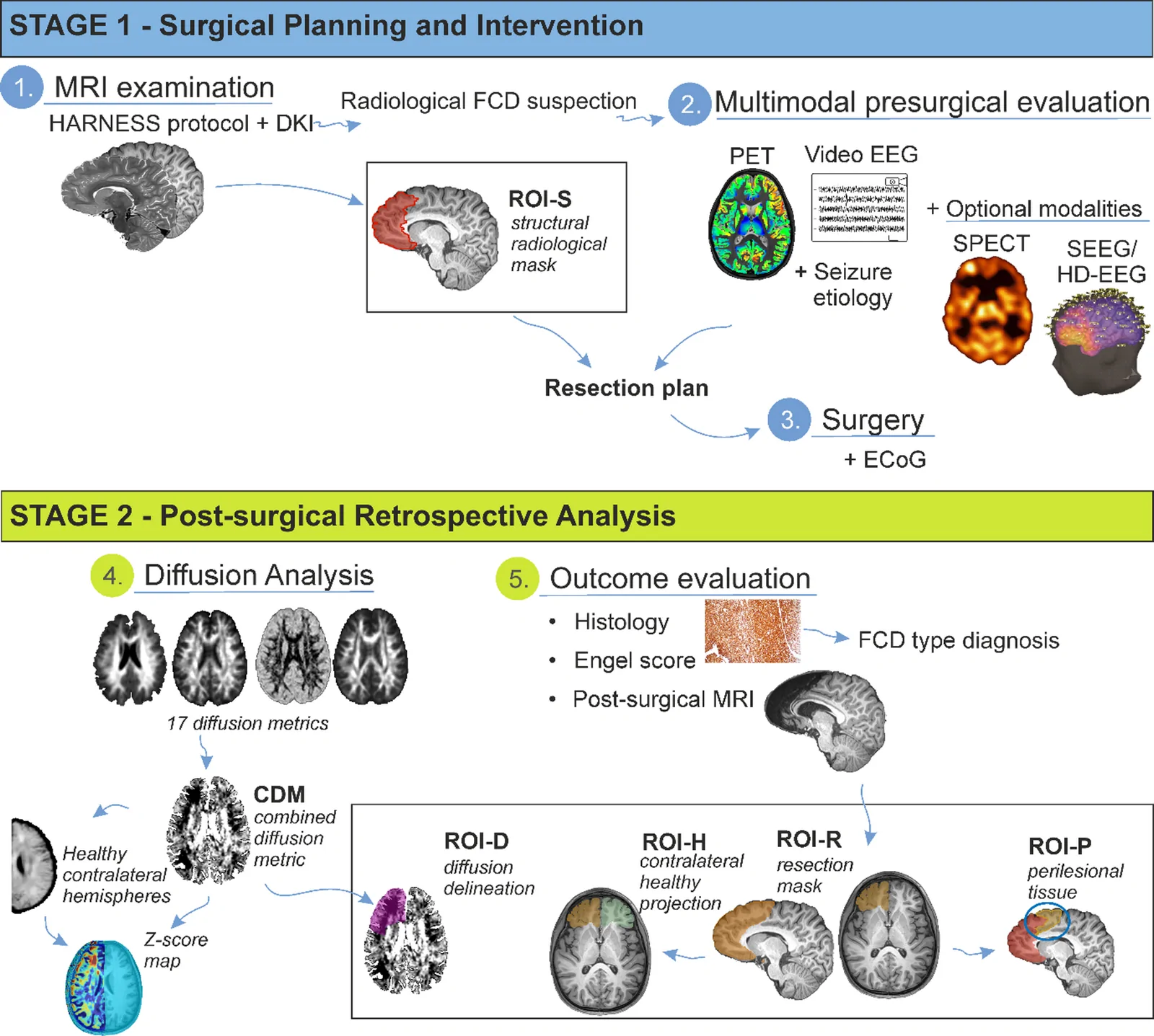
Study workflow and definition of regions of interest. Schematic overview of the analytical pipeline and ROI definition. FDG-PET and scalp video-EEG were performed in all patients as part of the presurgical evaluation, whereas HD-EEG, SPECT, and SEEG were used selectively to refine the surgical hypothesis. The defined ROIs included the structural lesion (ROI-S), the surgically resected region (ROI-R), its contralateral homologue (ROI-H), the perilesional tissue (ROI-P), and diffusion-derived abnormalities (ROI-D). These ROIs formed the basis for quantitative comparison of structural, diffusion, and metabolic data across the cohort.

### Patient cohort and clinical evaluation

Patients meeting the diagnostic criteria for FCD were consecutively enrolled from the cohort described above. Each patient underwent a standard preoperative evaluation consisting of clinical assessment, scalp video-EEG monitoring, high-resolution MRI, FDG-PET examination, and neuropsychological testing. Additional neuroimaging and electrophysiological studies, including intracranial EEG, were performed based on the discussion of each case at the multidisciplinary team meeting. All patients were postoperatively followed for at least 12 months, underwent postoperative MRI, and their resected brain tissue was subjected to comprehensive neuropathological analysis.

Inclusion criteria were:

- drug-resistant focal epilepsy with an MRI-visible lesion consistent with FCD;
- histological confirmation of FCD type I or II following surgical resection; and.
- completion of the epilepsy-specific MRI protocol, including post-surgical MRI follow-up.

Clinical data collected for each patient comprised age, sex, epilepsy duration, seizure type and frequency, laterality, and post-surgical outcome according to the Engel classification at least 12 months after surgery, and, when available, also reassessed at 24 months to report longer-term outcomes. (The histopathological subtype of FCD was determined in accordance with the current international classification^27,28^. Summary of demographic and clinical characteristics of the cohort are provided in Supplementary Table 1.

### Neuroimaging acquisition and pre-processing

#### MRI acquisition

All MRI examinations were performed at Motol University Hospital as part of the presurgical evaluation. Each patient underwent two scans: one pre-surgical and one post-surgical. Young or uncooperative paediatric patients (*n* = 10) were scanned under general anaesthesia following institutional safety protocols. Pre-surgical imaging was acquired on a 3 T Siemens Magnetom Vida with a 64-channel head coil using an epilepsy-optimized HARNESS protocol^23^. The protocol included structural and quantitative sequences: 3D T1-weighted (T1w), T2-weighted (T2w), and FLAIR images, and quantitative T1 mapping. Diffusion-weighted imaging (DWI) comprised 10 b0 images and diffusion weighting of b = 1000 and 3000 s/mm^2^, 20 gradient directions each. Detailed acquisition parameters are provided in Supplementary Tables 2 and the full protocol is available in our previous methodological publications^7,17,45^. Post-surgical MRI was performed on a 1.5 T Philips Achieva or 3 T Siemens Vida and included 3D T1w, T2w, FLAIR, and DWI sequences used for delineation of the resection cavity.

#### Image quality control and pre-processing

Pre-surgical and post-surgical images were co-registered and resampled to the native T1w image using SPM12 (rigid-body transformation, normalised mutual information cost function, 4th-degree B-spline interpolation). Bias-field correction and skull-striping was applied to all structural images. Tissue segmentation was performed on the T1w image using SPM12. For inter-subject comparison, images were further spatially normalized to MNI space. All scans underwent visual quality control and segmentation verification by experienced investigators (DK, MKy).

#### Definition of regions of interest

Five regions of interest (ROI) were defined in this study, as illustrated in Fig. 6. The lesion visible on structural MRI (ROI-S) was manually delineated on pre-surgical T1w, T2w, and FLAIR images by two experienced neuroradiologists (NP, MKy) in consensus. Lesions were delineated based on established MRI features of FCD (e.g. cortical thickening, grey-white matter blurring, abnormal signal, transmantle sign). For simplicity, this lesion will be referred to as the *structural lesion* or ROI-S throughout the manuscript. The resected region (ROI-R) was manually delineated on the pre-surgical T1w image by an experienced neuroscientist (RJ) through side-by-side visual comparison with post-surgical scans. This approach was chosen to account for post-operative brain shift and cavity deformation, as the anatomical boundaries of the resection may change after surgery; therefore, the resection margins were defined directly in the pre-surgical anatomical space. During delineation, gliotic tissue visible on post-operative imaging was not systematically included in the resection mask, as post-surgical gliosis may not necessarily represent functionally active or originally resected epileptogenic tissue. A conservative approach was therefore adopted to avoid overestimation of the resected volume. To enable intra-subject hemispheric comparisons, a contralateral homologue (ROI-H) was created by mirroring ROI-R across the midsagittal plane in MNI space. The perilesional region (ROI-P) was defined as the subtraction between ROI-R and ROI-S, representing tissue that was resected during surgery but did not show radiological abnormalities on structural MRI. Spatial overlap between ROI-S and ROI-R was quantified using the Dice similarity coefficient (Dice) to describe the correspondence between structural and surgical delineations.

### Multimodal clinical data for contextual validation

Multimodal presurgical data contributed primarily to the multidisciplinary definition of the surgical target and resected region. FDG-PET was additionally co-registered with MRI and assessed within the defined ROIs to provide spatial metabolic context for the diffusion findings.

#### Multimodal presurgical evaluation

Scalp video-EEG monitoring was performed in all patients as part of the standard presurgical evaluation in an epilepsy monitoring unit, in accordance with established international guidelines^46^. Additional modalities were applied selectively when further information was required to refine the surgical hypothesis. High-density EEG (HD-EEG) was acquired in selected patients (*n* = 15), stereo-EEG (SEEG) was performed in patients with non-localizing findings or lesions near eloquent cortex (*n* = 3), and SPECT was performed when standard presurgical data were insufficiently concordant or additional functional information was required (*n* = 2). Intraoperative electrocorticography (ECoG) was obtained in all patients using multiple strip electrodes placed over the lesion before and after resection to confirm interictal sources and completeness of their removal. These modalities contributed to multidisciplinary clinical interpretation and surgical planning, but were not included in the quantitative group-level diffusion analyses.

#### FDG-PET

All patients underwent pre-surgical FDG-PET acquired at specialized clinical centres using standard protocols^47^. Partial volume correction was applied based on T1w segmentation^40^. FDG-PET images were co-registered to the grey-matter segmentation derived from the T1w image using FastSurfer, ensuring alignment with the structural MRI space used for subsequent analyses. FDG-PET hypometabolism within each ROI was assessed by an experienced neuroscientist (RJ).

### Quantitative diffusion MRI analysis

#### Pre-processing of diffusion data

Diffusion-weighted data were pre-processed using MRtrix3^48^ and FSL frameworks. The pipeline included denoising^49^, Gibbs-ringing removal^50^, corrections for motion, eddy-current^51^, susceptibility distortion^52^, and bias-field^53^ and skull-striping. All processing steps followed recommended pipelines for advanced diffusion modelling.

#### Derivation of diffusion metrics

To comprehensively characterize microstructural abnormalities, we applied five complementary diffusion models. Each model provides distinct yet biologically meaningful information about tissue organization and composition.

- *DTI* assumes Gaussian diffusion and provides metrics such as mean diffusivity (MD) and fractional anisotropy (FA), reflecting overall tissue density and fibre coherence.
- *DKI* extends DTI to quantify non-Gaussian diffusion, yielding mean kurtosis (MK), kurtosis fractional anisotropy (KFA), and axial (Kax) and radial (Krad) kurtosis, sensitive to microstructural complexity and axonal integrity.
- *NODDI* estimates neurite density (NDI), orientation dispersion (ODI), and isotropic free-water fraction (FISO), capturing cellularity and extracellular space.
- *FBA* quantifies diffusion properties at the level of individual fibre populations using multi-tissue constrained spherical deconvolution, providing measures of fibre density (FD), cross-section (FC), and their combination (FDC).
- *DBSI* separates anisotropic and isotropic diffusion components, enabling estimation of axonal fibre fraction (AFF) and various isotropic diffusion fractions (IF): restricted (RIDF), nonrestricted (NRIDF), and hindered (HIDF);, distinguishing between cellular, extracellular, and free-water compartments.

Diffusion metrics were computed using standard toolboxes (Diffusion Kurtosis Estimator^54^, NODDI toolbox^14^, MRtrix3^55,56^, DBSI tool^25^. All tools were used with default parameters as described in the original publications. A summary of all diffusion metrics and their biological interpretation is provided in Supplementary Table 3.

#### Extraction of ROI-based metrics

All parametric maps were co-registered to the individual T1w image. Median metric values were extracted for each of the defined regions of interest: ROI-S, ROI-P, ROI-R, and ROI-H, using masks described above. For within-subject normalization, each metric value was also expressed relative to its contralateral homologue (ROI-H) to minimize inter-subject variability.

#### Construction of the combined diffusion metric (CDM)

##### Feature selection and standardization

To integrate complementary information from multiple diffusion models, fourteen voxel-wise metrics were included in the multivariate analysis: MD, FA, MK, KFA, FD, logFC, NDI, ODI, FISO, AFF, IF, HIDF, RIDF, and NRIDF. All parametric maps were normalized to MNI space. For each participant, voxel values were extracted from ROI-S and its contralateral homologue ROI-H. Outlier voxels were excluded using a Mahalanobis distance threshold exceeding the 95th percentile of the chi-square distribution with 14 degrees of freedom (number of included metrics)^57^. To ensure comparability across metrics, all voxel-wise values were standardized using Z-score normalization within each subject^58^. This procedure placed all diffusion metrics on a common scale, allowing the model to learn relative contributions rather than absolute magnitude differences.

##### Logistic regression model

A regularized logistic regression with LASSO (L1) penalty was used to identify the combination of diffusion metrics that best distinguished lesional from contralateral tissue^59^. LASSO regularization promotes sparsity by shrinking less informative coefficients toward zero, effectively performing feature selection and reducing model complexity. To prevent overfitting, leave-one-patient-out cross-validation was employed: for each held-out subject, the model was trained on all remaining patients, producing an independent set of regression coefficients^60^. Each voxel was weighted inversely to the number of voxels in its respective ROI, thereby preventing larger lesions from dominating the optimization process^61^. The resulting coefficients were used to compute the CDM as a linear combination of standardized diffusion features, weighted by their model-derived coefficients. The CDM thus represents a composite diffusion-based score optimized to maximize separability between lesional and healthy tissue.

##### Validation and visualization

Model performance was evaluated at both subject and group levels. At the individual level, Mann–Whitney U-tests compared CDM values between ROI-S and ROI-H, with effect sizes expressed as correlation coefficient R. Sensitivity analyses assessed model robustness by retraining classifiers using either the ROI-R versus its contralateral homologue or by restricting the training set to patients with histologically confirmed FCD type II.

Group-level voxel-wise Z-score maps were generated from spatially normalized CDM images to visualize the distribution of diffusion abnormalities across patients. All CDM maps were normalized to the MNI space and smoothed with a 3D Gaussian kernel (σ = 1 mm) to minimize residual misalignment. Contralateral (non-lesional) hemispheres were mirrored across the midsagittal plane and averaged to form a unified healthy hemisphere template representing normative diffusion characteristics. Ipsilateral hemispheres were processed identically and mirrored when necessary to ensure hemispheric alignment. Voxel-wise deviations from this reference template were expressed as normalized Z-score maps, indicating regions of diffusion abnormality.

### Histological analysis

Resected tissue samples were submitted for neuropathological examination. All specimens were formalin-fixed and paraffin-embedded. 2-µm H&E-stained sections were evaluated for cortical lamination, dysmorphic neurons, balloon cells, and the extent of gliosis by an experienced board-certified neuropathologist (MKo). The histopathological diagnosis was established according to the ILAE 2021 classification of FCD^27,28^.

Additionally, quantitative analysis of cellularity was performed in 12/19 patients; seven were excluded due to limited or fragmented diagnostic material. For each case, one H&E-stained section containing the epileptogenic lesion was selected. Cellularity was quantified in the subcortical white matter adjacent to the lesion using QuPath software with the StarDist algorithm for nuclear segmentation. Within each section, a single ROI corresponding to one high-power field (magnification 100×, area = 1.779 mm^2^) was defined, and all nuclei were automatically detected and manually verified. For correlation with imaging data, mean cellularity values, separately for white and grey matter, were compared with diffusion metrics derived from DBSI within the structural lesion (ROI-S) and its perilesional area (ROI-P).

### Radiological evaluation of structural and diffusion maps

The visibility of epileptogenic lesions was assessed visually by an experienced neuroradiologist (MKy) as an exploratory, clinically oriented evaluation intended to contextualise the interpretability of diffusion-derived maps. Each lesion was first delineated on structural MRI, defining the ROI-S during the initial reading. Subsequently, the radiologist reviewed individual diffusion maps and rated lesion conspicuity on a four-point scale (1 = not visible, 2 = mildly visible, 3 = visible, 4 = clearly visible; see Supplementary Fig. 2). These ratings were compared across modalities to evaluate the relative visual information contributed by each diffusion metric. After the introduction of the CDM map, the radiologist re-examined the previously defined lesion and adjusted its boundaries according to areas showing concordant or extended diffusion abnormalities. The resulting delineation was defined as the diffusion-derived lesion (ROI-D), representing tissue that exhibited diffusion abnormalities either overlapping with or extending beyond the structural lesion. The Dice similarity coefficient between ROI-D and the resection mask (ROI-R) was then calculated to quantify the improvement in spatial correspondence and to assess the added diagnostic value of the CDM. Examples of ROI-S and ROI-D overlays are shown in Supplementary Fig. 2.

### Statistical analysis

Statistical analyses were performed using MATLAB R2023a. Data normality was assessed using the Kolmogorov-Smirnov test, which indicated non-normal distribution across most variables. Consequently, two-tailed non-parametric tests (Wilcoxon signed-rank and Mann–Whitney U-tests) were applied throughout the analyses. To control for multiple comparisons across diffusion metrics, the Benjamini–Hochberg false discovery rate (FDR) correction was applied (q < 0.05). Effect sizes were expressed as correlation coefficients (R) derived from the standardized normal deviate (Z) and the number of observations (N), using the relation R = Z / √N, to emphasize practical over purely statistical significance.

Receiver operating characteristic (ROC) analysis was used to evaluate the diagnostic performance of the CDM. From ROC curves, the area under the curve (AUC), sensitivity, and precision were derived to determine optimal detection thresholds relative to the resection-defined reference region. In addition, individual ROC curves were inspected post hoc to identify patients whose ROC behaviour and AUC values deviated markedly from the remaining cohort. These cases were not excluded from the primary analysis but were additionally omitted in a sensitivity analysis to assess the robustness of the group-level ROC estimate. After identifying these cases, we examined whether they differed from the remaining patients in potential confounding variables, including age, sex, FCD subtype, and lesion volume.

Unless otherwise specified, data are reported as median values with interquartile ranges (IQR) in parentheses. Statistical significance was set at α = 0.05, and significance levels in figures are indicated as *p* < 0.05 (*), *p* < 0.01 (**), *p* < 0.001 (***), or non-significant (ns).

The primary analytical objective was to evaluate whether the CDM improves delineation of epileptogenic tissue in FCD by increasing spatial correspondence with the resected region. Additional analyses were performed to further validate CDM performance, including voxel-wise Z-score and ROC approaches, while ROI-based comparisons of individual diffusion metrics were used to characterize underlying diffusion alterations. Visual assessment, multimodal comparison with FDG-PET, and histological correlations were included to provide complementary biological and clinical context.

During the preparation of this work, the author(s) used ChatGPT to improve the clarity and readability of the manuscript. The author(s) take full responsibility for the content of the published article.

## Supplementary Information

Below is the link to the electronic supplementary material.

Supplementary Material 1

## Data Availability

The data that support the findings of this study are available from the corresponding author upon reasonable request. Due to the sensitive nature of the clinical data and applicable data protection regulations, the data are not publicly available. Access may be granted to qualified researchers subject to institutional approval and compliance with relevant ethical and legal requirements.
