# Supplementary material for: Beyond structural MRI: combined diffusion metric improve delineation of epileptogenic tissue in focal cortical dysplasia
Source: Sci Rep. 2026 Jun 9;16:26247. doi: 10.1038/s41598-026-57292-w (PMC13493953; doi:10.1038/s41598-026-57292-w)
Supplement: Supplementary file 1 [file 41598_2026_57292_MOESM1_ESM.pdf]

Supplementary material

Supplementary Table 1. Demographic, clinical, and surgical characteristics of patients included in the study.

ID	Age at MRI	Sex	Epilepsy duration [Yrs]	Seizure frequency [/day]	Status epilepticus*	Types of seizures	FCD type	Localization	Laterality	FDG-PET lesion	FDG-PET perilesion	ECoG – presurgery	Resection volume [cm³]	Resection extent [0–1]	Dice [ROI-S × ROI-R]	ECoG – postsurgery	Outcome [Engel]‡
S1	3Y	F	3	0.3	-	Tonic - clonic	II	Parietal	R	full	partial	REDs, poly	8,24	0,8	0.47	normal	1A (1A)
S2	1Y, 5M	M	1	3	-	Epileptic spasms	I	Parietal	L	partial	normal	lowV IED	27,76	0,99	0.42	lowV IED	3A (3A)
S3	2Y, 1M	M	1	3-5	-	Behavioural arrest, myoclonic	II	Parietal, Temporal	R	full	full	normal	9,64	0,68	0.01	normal	1A (1A)
S4	9Y	M	9	20	35 days	Sensory (abdominal sensations, noise), emotional, automatisms (hands), clonic (hands)	II	Frontal, Temporal, Parietal	L	none	none	REDs, poly	133,07	0,91	0.49	lowV IED	3A (1A)
S5	8Y	F	5	1	-	Emotional (fright), autonomic (hyperventilation, tachycardia), automatisms (OAA, restlessness), aphasia	II	Frontal	L	full	partial	REDs, poly	7,56	0,89	0.36	lowV IED	1A (1A)
S6	5Y	F	1	3	6 days	Epileptic spasms	I	Frontal, Temporal	R	partial	partial	lowV IED	105,5	0,99	0.69	normal	1A (1A)
S7	5Y	F	2	20-30	During MRI	Myoclonic (right hand), tonic (right hand)	II	Frontal	L	full	minimal	REDs, poly	6,35	0,78	0.29	normal	1A (1A)
S8	17Y	F	12	1	-	Automatisms	I	Frontal	R	none	minimal	highV IED, Burst suppression.	99,56	0,94	0.72	normal	1A (1A)
S9	0Y, 6M	F	0.4	2	-	Behavioural arrest, autonomic (flushing)	II	Frontal	L	NA	NA	lowV IED	19,3	0,81	0.67	normal	1A (1A)
S10	55Y	M	26	4	9 months	cognitive aura, behaviour arrest, falls, FBTCS	II	Frontal	R	partial	full	REDs, poly	16,89	NA	0.45	normal	3A (3A)
S11	6Y	F	1	4	-	Sensory (tingling L thorax), tonic (L hand, L mouth corner), gelastic, L hand plegia, clonic (L hand/leg), confusion	II	Frontal	R	partial	partial	lowV IED	13,37	0,85	0.15	normal	1A (1A)
S12	17Y	F	13	0	-	Behavioural arrest, tonic (left hand), sensory (left hand paresthesia), atonic/tonic, clonic (left hand), hemiparesis, tonic–clonic (left side), FBTCS	II	Frontal, Parietal	R	full	minimal	highV IED, Burst suppression.	2,9	0,72	0.48	lowV IED	1A (1A)
S13	17Y	F	14	8-15	-	Behavioural arrest, tonic (eyes/head version, left mouth corner), grunting, horizontal eye deviation,	II	Frontal	R	full	partial	highV IED, Burst suppression.	2,82	0,93	0.53	lowV IED	1A (1A)

						tachypnoea, hypermotor activity, FBTCS											
S14	3Y	F	2.5	5-7	35 months	Frightened face, hypermotor automatisms (crawling/blinking), eye deviation (L→R), tonic version (eyes/head R), clonic (right mouth corner)	II	Occipital	L	full	full	lowV IED	13,53	0,78	0.11	normal	1A (1A)
S15	29Y	F	16	5	-	sensory aura - feeling of disturbed time and space, behaviour arrest	II	Frontal	L	partial	partial	normal	9,15	NA	0.21	normal	1A
S16	13Y	F	0.5	10	-	FBTCS, behavioural arrest with upward eye deviation	II	Frontal	R	Partial Hyper	full	REDs, poly	12,38	0,75	0.18	REDs, poly	1A
S17	2Y	M	1	3	-	Left hand plegia, clonic movements (left mouth corner)	II	Parietal	R	full	partial	REDs, poly	12,05	0,96	0.69	normal	1A
S18	17Y	F	15	0	-	Behavioural arrest with horizontal eye deviation, confusion, FBTCS	II	Parietal	R	full	none	normal	9,35	0,89	0.67	normal	1A
S19	11Y	M	17	0.3	-	FBTCS, behavioural arrest, vestibular and experiential aura	II	Parietal	R	full	partial	highV IED, Burst suppression	9,24	NA	0.23	normal	1A

*Time between last reported status epilepticus and MRI

‡Engel evaluation 12 months post-surgery (24 months if available)

FDG-PET: visually evaluated degree of cortical hypometabolism (none, minimal, partial, full).

ECoG: description of epileptiform activity pattern obtained during pre-surgical or intraoperative monitoring.

IED (interictal epileptiform discharges), poly (polyspike discharges), REDs (repetitive epileptiform discharges).

Supplementary Table 2. Acquisition parameters of MRI data

Sequence	Type	TR (ms)	TE (ms)	TI (ms)	FA (deg)	Averaging	Acquisition matrix (mm)	Voxel size (mm)	Acceleration factor	Duration (min:sec)
Pre-surgical examination										
T1w	3D MP-RAGE	2400	2.29		8	1	0.8x0.8x0.8	0.4x0.4x0.8	GRAPPA - 2	6:47
T2w	3D SPACE	3200	5.72		Variable*	1.8	0.8x0.8x0.8	0.4x0.4x0.8	CAIPIRINHA - 3	6:35
FLAIR	3D SPACE	6000	350	1900	Variable*	1.8	1x1x0.9	0.5x0.5x0.9	CAIPIRINHA - 4	8:02
qT1	Gradient echo	15	2		4 and 23	1	1x1x1	0.5x0.5x1	GRAPPA - 2	8:45
DWI**	EPI	5300	97			1	2x2x2	2x2x2	SMS - 3	4:42
Post-surgical examination										
T1w	3D TFE	7.1	3.2	950	8	1	1x1x1	1x1x1	SENSE (AP) 1, (RL) 2	04:59
T2w	3D TSE	2800	280		90	2	1x1x1	0.78x0.78x0.5	CS SENSE - 6	04:04
FLAIR	TSE	9000	140	2450	90	2	0.9x1.25x4	0.9x0.9x4	SENSE 1.5	02:15
DWI***	EPI	10233	86		90	2	2x2x2	1.8x1.8x2	SENSE 2	05:38

*TSE with variable flip angle of refocusing RF pulses (for detail see Katherine T. Scott. SPACE: An Innovative Solution to Rapid, Low SAR, T2-Weighted Contrast in 3D Spin Echo Imaging. Siemens Medical Solutions Published online 2009.); ** b 1000 and 3000 s/mm2 in 20 directions each + 5 b0 images with posterior- anterior phase encoding direction and 5 b0 images in anterior–posterior direction; *** b 1000 in 15 directions + 1 b0

TR (repetition time), TE (echo time), TI (inversion time), FA (flip angle)

Supplementary Table 3. Overview of diffusion metrics used in this study

Metric name	Abbreviation	Model	Interpretation
Mean Diffusivity	MD	DTI	Tissue density and integrity
Fractional Anisotropy	FA	DTI	Tracts organization
Mean Kurtosis	MK	DKI	Microstructure complexity
Kurtosis Fractional Anisotropy	KFA	DKI	Tracts organization
Axial Kurtosis	Kax	DKI	Axonal integrity
Radial Kurtosis	Krad	DKI	Axonal integrity
Orientation Dispersion Index	ODI	NODDI	Orientation of neurites
Neurite Density Index	NDI	NODDI	Density of neurites
Isotropic Volume Fraction	FISO	NODDI	Amount of extracellular free water
Fiber Density	FD	FBA	Axonal integrity
Fiber Cross-Section	FC	FBA	Morphology of the fibre
Combined FD and FC	FDC	FBA	Overall health of fibre population
Axonal Fiber Fraction	AFF	DBSI	Axonal density and integrity
Restricted Isotropic Diffusion Fraction	RIDF	DBSI	Cellular density
Hindered Isotropic Diffusion Fraction	HIDF	DBSI	Extra-axonal space density
Non-Restricted Isotropic Diffusion Fraction	NRIDF	DBSI	Free water density
Isotropic Fraction	IF	DBSI	Extracellular contribution

Supplementary Table 4. Individual radiological ratings of lesion visibility across MRI and diffusion metrics.

**Subject**	**S1**	**S2**	**S3**	**S4**	**S5**	**S6**	**S7**	**S8**	**S9**	**S10**	**S11**	**S12**	**S13**	**S14**	**S15**	**S16**	**S17**	**S18**	**S19**
FCD type	II	I	II	II	II	I	II	I	II	II	II	II	II	II	II	II	II	II	II
**metric**	**lesion visibility score**																		
T1w	4	3	4	4	3	3	4	3	3	4	2	4	4	4	3	4	4	4	4
T2w	4	3	2	3	4	3	4	3	4	3	2	4	4	4	3	4	4	4	4
FLAIR	4	3	2	3	3	3	4	4	4	3	2	3	4	4	3	4	4	4	4
DWI	2	2	1	2	1	1	2	1	2	1	1	1	2	2	1	2	3	2	3
MD	1	2	1	2	3	2	2	1	2	1	1	2	2	1	1	1	3	2	1
FA	2	1	1	2	1	2	2	2	2	2	1	2	2	1	1	1	2	3	1
MK	3	1	1	2	1	1	2	1	2	2	1	2	2	1	1	1	3	2	1
KFA	1	1	2	2	2	1	1	1	2	2	1	3	2	1	1	2	2	2	1
ODI	2	1	2	2	2	2	3	1	2	2	1	2	2	1	1	2	2	3	1
NDI	2	1	1	2	3	2	2	1	3	2	1	2	2	1	1	1	2	2	2
FISO	1	1	2	2	1	2	1	1	1	2	1	1	2	1	1	2	2	1	1
FD	2	2	1	2	1	2	2	1	2	2	1	2	2	1	1	2	1	2	1
logFC	1	1	1	2	2	2	1	1	1	1	2	2	2	2	1	2	1	2	2
FDC	2	1	1	2	1	2	2	1	2	2	1	2	2	1	1	2	1	1	2
AFF	2	2	1	2	1	3	2	2	2	2	1	2	2	2	2	2	3	2	2
HIDF	3	3	1	2	1	2	3	1	2	1	1	1	2	1	1	1	3	3	1
IF	3	2	1	2	2	2	3	1	2	1	1	2	2	2	1	2	3	3	2
RIDF	2	2	2	2	2	2	2	1	3	2	1	1	2	2	1	2	3	3	2
NRIDF	2	1	2	2	2	1	2	1	2	2	1	1	1	1	1	1	3	2	1

Lesion visibility scores (1–4) assigned by an experienced neuroradiologist (MK) for each patient (S1–S19) across structural MRI sequences (T1w, T2w, FLAIR) and diffusion-derived maps (DTI, DKI, NODDI, FBA, DBSI). Higher scores indicate greater lesion conspicuity. Rows correspond to individual metrics, columns to patients, with the FCD subtype (I or II) indicated in the second row. The table illustrates considerable inter-patient and inter-metric variability in visual detectability, with structural MRI consistently outperforming single diffusion metrics.

Supplementary Table 5. Statistical comparison of diffusion metrics between the structural lesion and contralateral homologue

	**ROI-H vs ROI-S**	**ROI-H vs ROI-P**	**ROI-S vs ROI-P**
T1w	0.051	0.099	**0.022**
T2w	**0.044**	0.549	0.153
FL	**0.016**	0.683	**0.022**
DWI	0.453	0.160	0.077
FA	**0.044**	1.000	**0.025**
KFA	**0.016**	0.860	**0.022**
MD	0.276	0.265	0.074
MK	0.610	0.265	0.189
Krad	0.453	0.352	0.153
Kax	0.695	0.187	0.156
AFF	0.077	0.549	**0.034**
HIDF	0.102	0.549	0.063
IF	0.084	0.549	**0.041**
RIDF	0.669	0.265	0.172
NRIDF	0.717	0.883	0.872
ODI	0.221	0.549	0.105
NDI	0.717	0.160	0.095
FISO	0.238	0.549	0.519
FD	**0.020**	0.099	**0.022**
FDC	**0.020**	0.099	**0.023**
logFC	**0.044**	0.230	**0.041**

False-discovery-rate (FDR) corrected *p* values for all diffusion metrics derived from DTI, DKI, NODDI, FBA, and DBSI models. Each value reflects the within-subject comparison between the structural lesion (ROI-S) and its contralateral homologue (ROI-H). Metrics with $q < 0.05$ were considered significant and are highlighted in bold. The table shows that only a limited subset of parameters: fractional anisotropy (FA), kurtosis fractional anisotropy (KFA), fibre density (FD), and fibre cross-section (FC), exhibited significant differences after FDR correction.

Supplementary Figure 1. Examples of visual radiological scoring of lesion visibility

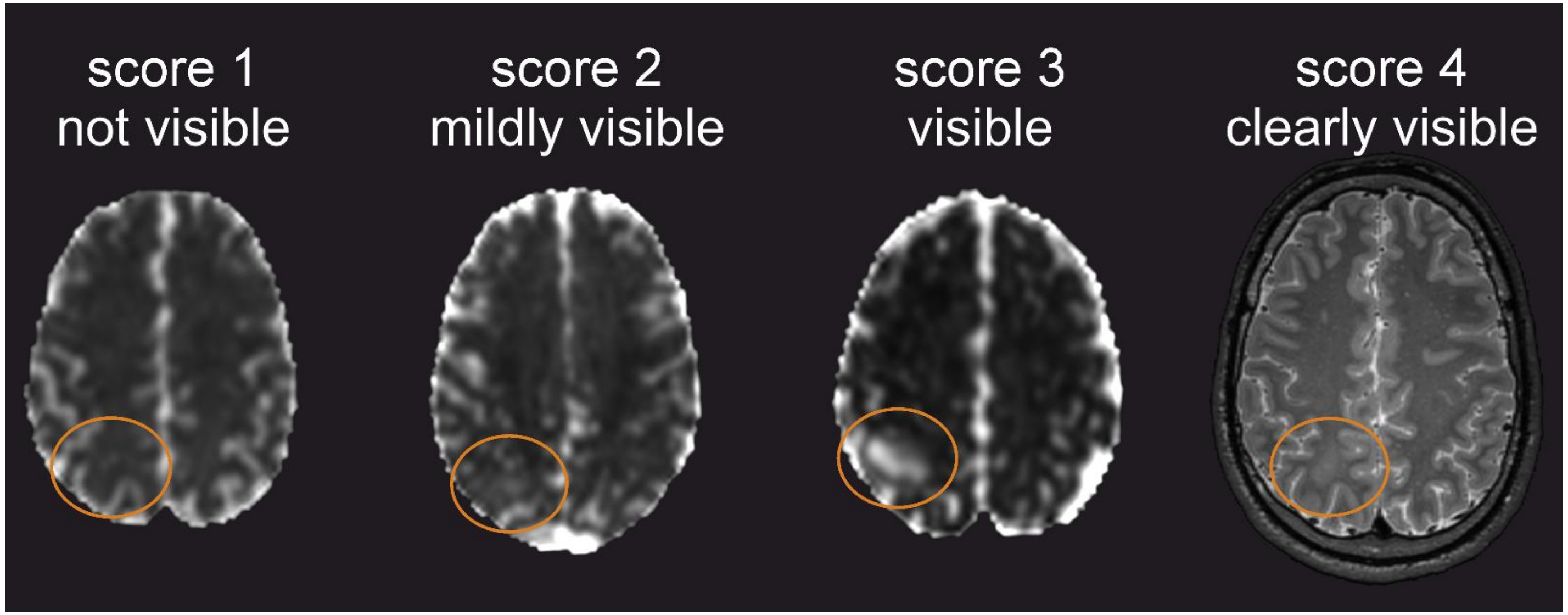

Representative examples illustrating the subjective visual scoring scale used to assess epileptogenic lesion visibility across different diffusion metrics. Scores 1–3 correspond to lesions evaluated on Mean Diffusivity maps in three different patients. Score 4 was achieved only on structural MRI and is therefore shown on a T2-weighted image. In all cases, the epileptogenic lesion was located in a similar region, highlighted by an orange circle.

Supplementary Figure 2. Definition of diffusion-derived lesion (ROI-D) based on radiological re-evaluation using the combined diffusion metric (CDM).

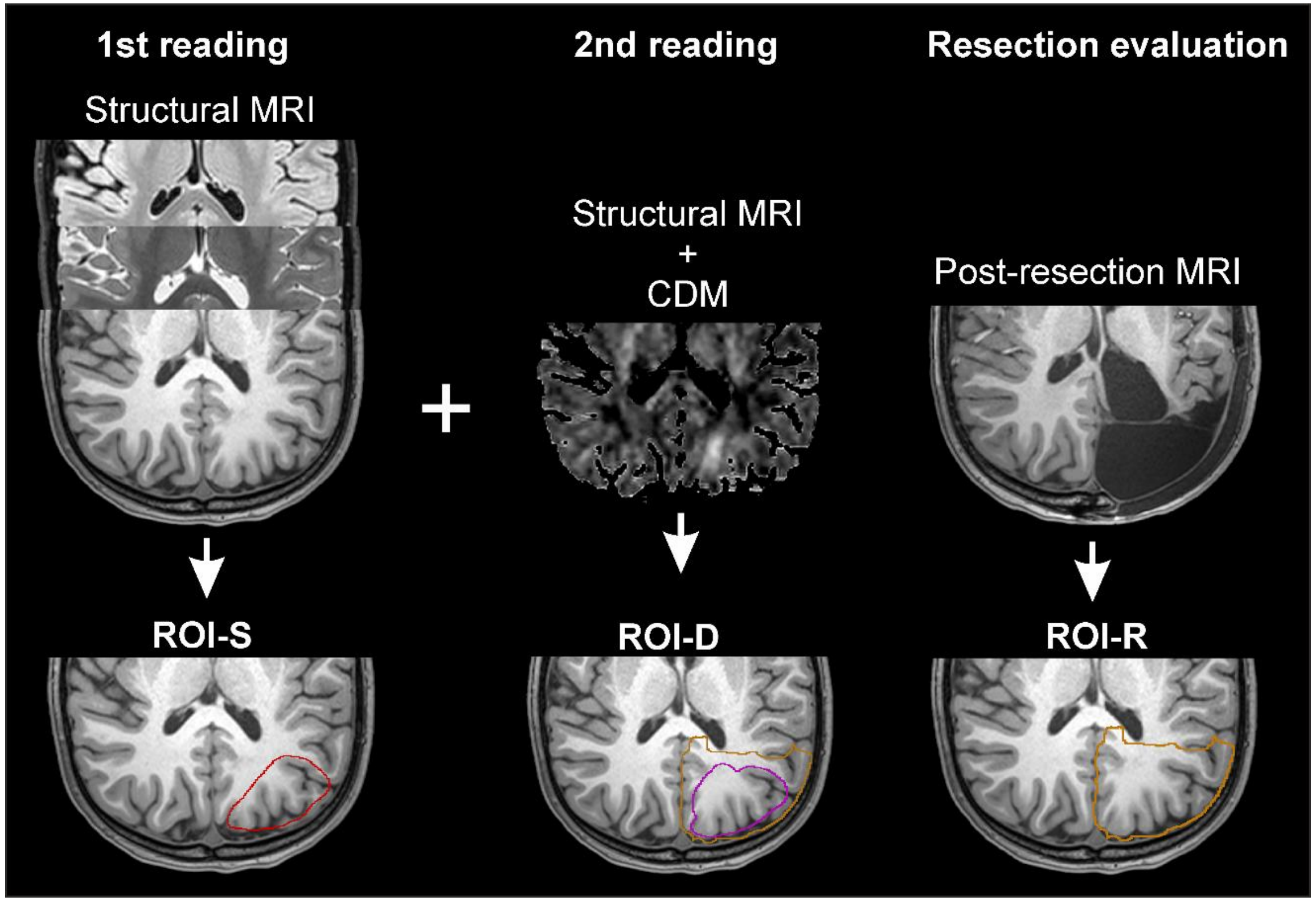

The figure illustrates the radiological workflow leading to the definition of the diffusion-derived lesion (ROI-D). Left column shows the structural lesion (ROI-S) delineated on conventional MRI during the first reading. Middle column displays the Combined Diffusion Metric (CDM) map revealing diffusion abnormalities partially overlapping and extending beyond the structural lesion. Right column shows the spatial relationship between ROI-D and the resection mask (ROI-R) in the postoperative scan. **This figure illustrates how integration of the CDM facilitated identification of diffusion abnormalities beyond the radiologically visible lesion and supported refinement of the epileptogenic tissue delineation.**

Supplementary Figure 3. Histological and diffusion differences between FCD subtypes.

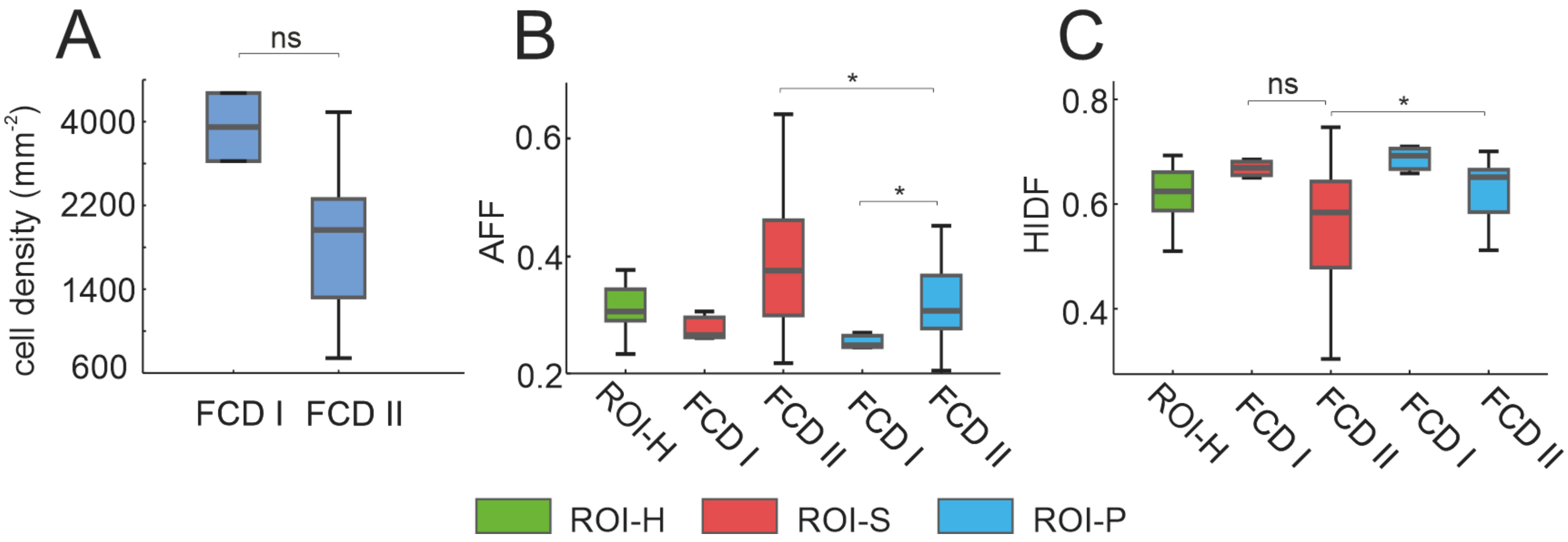

Comparison of histological cellularity and DBSI-derived metrics between FCD I and FCD II. (**A**) Cell density in FCD II tended to be lower than in FCD I, although this trend did not reach statistical significance. (**B**) The axonal fibre fraction (AFF) was higher in FCD II, consistent with more preserved fibre organisation. (**C**) The hindered isotropic diffusion fraction (HIDF) showed lower median values in FCD II than in FCD I, but the difference was not significant. **Together, these findings suggest modest subtype-related microstructural differences that follow the expected direction but require confirmation in a larger cohort.**

Supplementary Figure 4. Weights of the logistic regression model used to construct the combined diffusion metric (CDM)

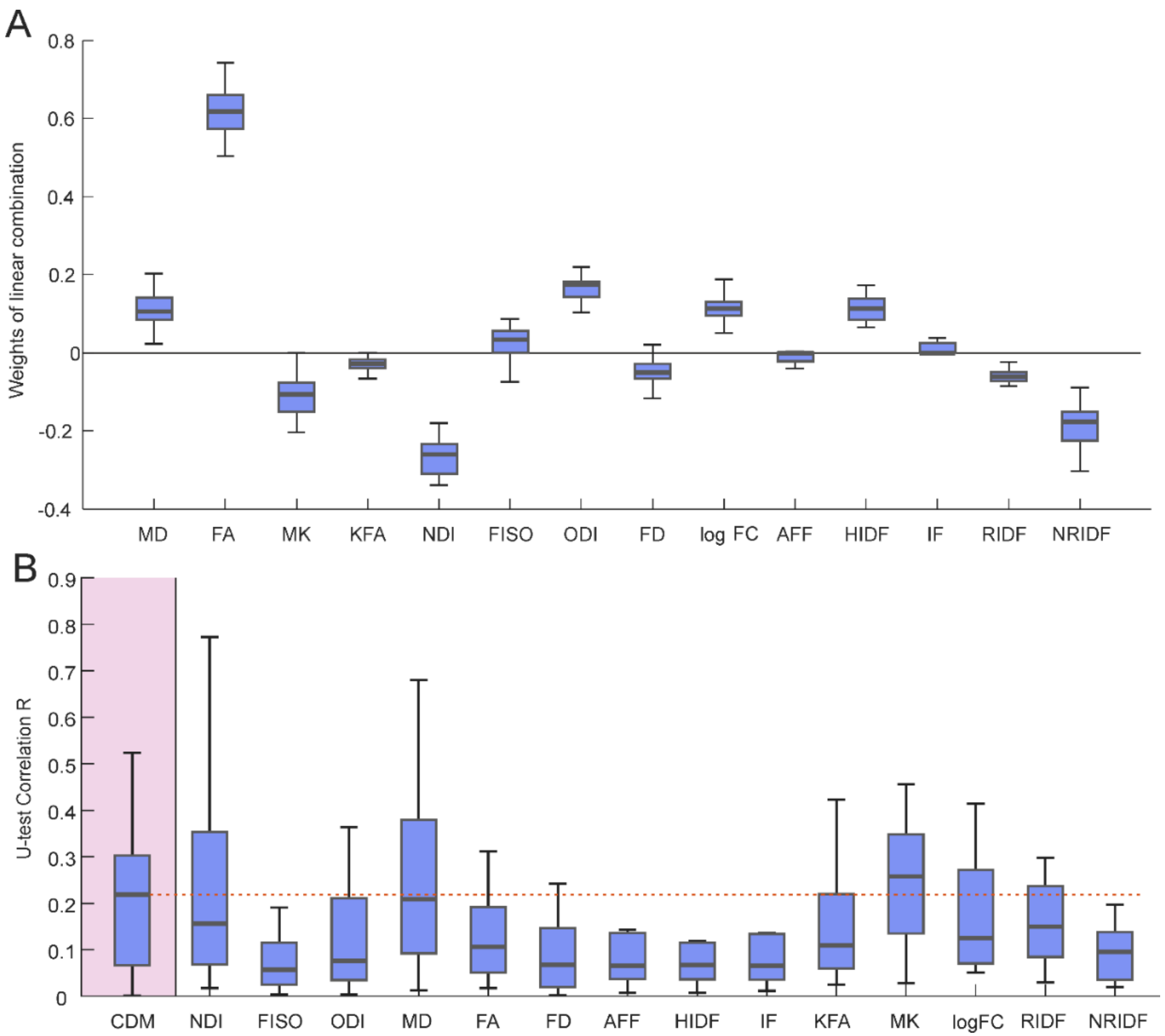

(**A**) Bar graph showing the relative contribution (median ± IQR) of each diffusion parameter in the logistic regression trained to maximise discrimination between the structural lesion (ROI-S) and its contralateral homologue (ROI-H). The highest positive weights were assigned to FA, ODI, and logFC, while negative coefficients were associated mainly with NDI, NRIDF, and MK. These weights were used to compute the voxel-wise Combined Diffusion Metric shown in Figure 8 of the main text. (**B**) Correlation coefficient R derived from the Mann–Whitney U-test for individual diffusion metrics and the CDM. The analysis quantifies the contrast between pathological tissue (ROI-S) and its projected healthy counterpart (ROI-H), where higher R values indicate stronger differentiation between ROIs. CDM demonstrates higher contrast compared to most individual diffusion metrics, indicating improved sensitivity to tissue alterations. Only MD and MK exhibit comparable R values to CDM.
